# Auto-Induction in Oral Esketamine Treatment for Treatment-Resistant Depression: An Exploratory Study

**DOI:** 10.3390/ph18050627

**Published:** 2025-04-25

**Authors:** Jolien K. E. Veraart, Cornelis F. Vos, Nieko C. Punt, Dylan Visser, Mireille A. Wessels, Sanne Y. Smith-Apeldoorn, Jeanine Kamphuis, Robert A. Schoevers, Daan J. Touw

**Affiliations:** 1 Department of Psychiatry, University Medical Center Groningen, University of Groningen, 9713 GZ Groningen, The Netherlands; j.k.e.veraart@umcg.nl (J.K.E.V.); s.y.apeldoorn@umcg.nl (S.Y.S.-A.); j.kamphuis01@umcg.nl (J.K.); r.a.schoevers@umcg.nl (R.A.S.); 2 Department of Psychiatry, PsyQ Haaglanden, Parnassia Psychiatric Institute, 2552 DH The Hague, The Netherlands; 3 Antes, Parnassia Group, 3066 SG Rotterdam, The Netherlands; n.vos@anteszorg.nl; 4 Department of Clinical Pharmacy and Pharmacology, University Medical Center Groningen, University of Groningen, 9713 GZ Groningen, The Netherlands; punt@medimatics.nl (N.C.P.); d.visser.15@student.rug.nl (D.V.);; 5 Medimatics, 6229 HR Maastricht, The Netherlands; 6 School of Behavioral and Cognitive Neurosciences, University of Groningen, 9701 BA Groningen, The Netherlands

**Keywords:** esketamine, treatment-resistant depression, pharmacokinetics, auto-induction

## Abstract

**Background:** Esketamine is a rapidly acting antidepressant with robust efficacy in treatment-resistant depression (TRD). Diminishing therapeutic effects and attenuated side effects have been reported after long-term use. This study aimed to investigate its long-term pharmacokinetics and factors that may contribute to reduced efficacy over time in patients with TRD by evaluating the potential role of auto-induction. **Methods:** Pharmacokinetic data were collected from 18 patients receiving oral esketamine for six weeks. A pharmacokinetic model was developed to predict esketamine and noresketamine plasma concentrations. Observed esketamine and noresketamine plasma concentrations were compared to model-predicted concentrations to assess deviations suggestive of auto-induction. **Results:** On day 39, plasma concentrations of esketamine and noresketamine were 59% and 35% lower than predicted, respectively, indicative of auto-induction of CYP3A4 and CYP2B6. **Conclusions:** Auto-induction appears to occur in oral esketamine treatment, which may contribute to reduced therapeutic efficacy and side effects in long-term treatment. Identifying auto-induction as a mechanism of tolerance potentially has important clinical implications. Further studies are warranted to confirm these findings and evaluate strategies to maintain therapeutic efficacy.

## 1. Introduction

Esketamine has recently gained approval as a novel treatment option for treatment-resistant depression (TRD), with its use rapidly increasing worldwide [1,2,3]. As a non-selective, non-competitive antagonist of the glutamatergic *N*-methyl-D Aspartate (NMDA) receptor, esketamine demonstrates robust antidepressant effects [4]. Next to its registered intranasal administration, esketamine is available in various forms for therapeutic use, including as liquid or capsules for oral ingestion [5]. The oral route of administration is of particular interest due to its ease of use, cost-effectiveness, and potential for at-home administration, provided that adequate monitoring is in place. Meta-analyses of randomized controlled trials (RCTs) on oral (es)ketamine and real-world data demonstrate significant antidepressant effects, although larger RCTs with solid study designs are warranted [6,7].

When administered orally, esketamine undergoes extensive first-pass metabolism, converting esketamine primarily into its main metabolite, noresketamine, through the action of Cytochrome P-450 (CYP) enzymes, most notably CYP2B6 and CYP3A4 [2,8]. Additional enzymes involved in this process are CYP2A6, CYP3A5, and CYP2C19. Noresketamine is subsequently metabolized into dehydronoresketamine (DHNK), primarily via CYP2B6, and into hydroxynoresketamine (HNK). The relative antidepressant effects of esketamine metabolites remain unknown [4]. Because of its extensive first-pass metabolism, the bioavailability of orally administered esketamine is 10% to 20% [2].

During esketamine therapy, long-term maintenance treatment is often required due to the risk of relapse after discontinuation [9]. However, it has been described that patients undergoing esketamine therapy may experience reductions in both efficacy and adverse effects over time, even in case of continued treatment with the same dose and frequency. In an interim analysis of the SUSTAIN-3 study, which is the study with the longest follow-up duration of intranasal esketamine for TRD to date, findings are reported from patients with an average treatment duration of 31.5 months [10]. Among the 1110 participants, seven participants discontinued due to worsening depression (reported as an adverse event), and 49 participants discontinued due to lack of efficacy. This indicates that 5.0% of the initial responders were unable to sustain antidepressant effects during the maintenance phase. A systematic review on the efficacy, safety, and tolerability of maintenance ketamine treatment for depression found that the most frequently reported reasons for discontinuation of maintenance treatment were (partial) relapse or worsening of depressive symptoms [11]. In addition to the antidepressant effects, multiple studies investigating intranasal and intravenous esketamine for TRD have found that dissociative symptoms and perceptual changes attenuate with repeated dosing [9,12,13,14].

Several factors may contribute to this reduction in antidepressant efficacy and side effects over time, one of which is the development of tolerance to esketamine. Tolerance is commonly observed and well-described in individuals who use ketamine frequently for recreational purposes [15]. A potential underlying mechanism for the development of tolerance could be auto-induction, a process whereby a drug enhances the expression of CYP enzymes involved in its own metabolism, leading to reduced plasma concentrations with repeated use [16]. Auto-induction has been reported for CYP2B6 and CYP3A4 with other substrates [17], but its occurrence in treatment with esketamine remains unclear so far.

The aim of this study was to investigate whether auto-induction occurs during oral esketamine treatment in patients with TRD. To this end, we assessed and compared esketamine and noresketamine plasma concentrations over time. In the case of auto-induction, we expected lower-than-predicted esketamine and noresketamine plasma concentrations after prolonged use of oral esketamine. Identifying auto-induction as a contributing factor to reduced effectiveness and diminished adverse effects over time could have important implications for optimizing dosing regimens in clinical practice.

## 2. Results

### 2.1. Study Population

For the current analysis, 18 participants were included, 9 females and 9 males. The mean age of the study group was 51.9 years (SD 10.9). None of the patients used concomitant medications causing drug–drug interactions with esketamine throughout the treatment period.

### 2.2. Esketamine and Noresketamine Plasma Concentrations

At day five of treatment, multiple esketamine and noresketamine samples were collected. The peak esketamine plasma concentration was, on average, 15.0 ng/mL (SD 7.4), median 14.1 ng/mL. The peak noresketamine plasma concentration was, on average, 104.4 ng/mL (SD 22.1), median 99.2 ng/mL.

**Figure 1:**
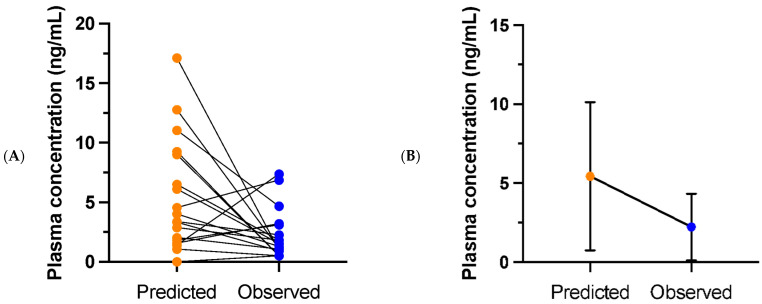
Predicted and observed esketamine plasma concentrations on day 39 of treatment for (**A**) individual patients and (**B**) presented as means with standard deviations (*n* = 18).

**Figure 2:**
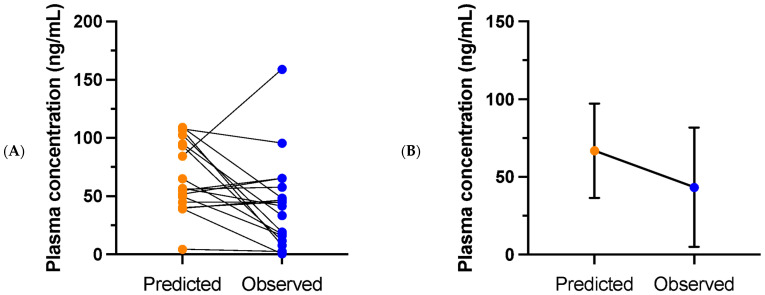
Predicted and observed noresketamine plasma concentrations on day 39 of treatment for (**A**) individual patients and (**B**) presented as means with standard deviations (*n* = 18).

At day 39, plasma concentrations were collected at variable time intervals post-dose. The predicted and observed esketamine and noresketamine plasma concentrations are shown in Figure 1 and in Figure 2, respectively. For esketamine, the observed plasma concentration was, on average, 2.2 ng/mL (SD 2.1), median 1.4 ng/mL, while the mean predicted concentration was 5.4 ng/mL (SD 3.7), median 3.7 ng/mL. As a result, the observed esketamine plasma concentration was, on average, 59% lower than the predicted value. The mean difference between the predicted and observed concentrations was 2.5 ng/mL (SD 5.9), median 1.4 ng/mL. The difference between the predicted and observed esketamine plasma concentrations was normally distributed: Shapiro–Wilk test: W (18) = 0.96, *p* = 0.59. A paired *t*-test demonstrated that the observed concentration was significantly lower than the predicted concentration, t (17) = 2.5, *p* = 0.01 (one-sided).

For noresketamine, the mean observed plasma concentration at day 39 was 43.2 ng/mL (SD 38.5), median 43.2 ng/mL, while the mean predicted concentration was 66.8 ng/mL (SD 30.3), median 56.4 ng/mL. Consequently, the observed noresketamine concentration was, on average, 35% lower than the predicted value. The mean difference between the predicted and observed concentrations was 23.5 ng/mL (SD 43.5), median 13.7 ng/mL. The difference between the predicted and observed noresketamine plasma concentrations was normally distributed: Shapiro–Wilk test: W (18) = 0.95, *p* = 0.46. A paired *t*-test showed that the observed noresketamine concentration was significantly lower than the predicted concentration, t (17) = 2.3, *p* = 0.02 (one-sided).

A post hoc Pearson correlation test revealed that participants with the greatest difference between predicted and observed esketamine concentrations also had the greatest difference between predicted and observed noresketamine concentrations: r (18) = 0.85, *p* ≦ 0.001 (two-tailed).

## 3. Discussion

Using data from 18 patients receiving oral esketamine for six weeks, we developed a population pharmacokinetic model describing the pharmacokinetics of oral esketamine and used this model to explore the occurrence of auto-induction in treatment with oral esketamine. On day 39 of treatment, we observed significantly lower-than-predicted esketamine and noresketamine plasma concentrations, suggesting the presence of auto-induction. Moreover, a correlation was found between the decrease in predicted and observed concentrations of esketamine and noresketamine, indicating that the lower-than-expected plasma concentrations are not random, but may be linked by the same enzymatic process.

While the occurrences of reduced efficacy and side effects during long-term esketamine treatment for depression have been well documented, the literature currently provides no information on auto-induction as a potential underlying mechanism. Several studies, however, did examine the auto-inductive properties of racemic ketamine. Previous findings provide evidence for inductive effects of ketamine on both CYP3A4 and CYP2B6. In rodents, daily ketamine administration has been shown to increase the activities of CYP2B6 and CYP3A4 [18,19,20,21]. Marietta et al. report that pretreatment with 40 mg/kg of intraperitoneal ketamine twice daily for three days resulted in lower ketamine plasma levels and a decreased duration of anesthesia after the administration of 30 mg/kg intravenous ketamine when compared to non-pretreated controls [18]. Livingston and Waterman describe a more rapid decrease in plasma and brain levels of ketamine and norketamine after an injection of 75 mg/kg intraperitoneal ketamine in rats that were pretreated with 40 mg/kg intraperitoneal ketamine daily for ten days, compared to a control group [19]. These effects were associated with a decrease in sleeping time in the pretreated rats. In addition, they demonstrated an increased rate of metabolism of ketamine by the liver tissue of pretreated rats. Chan et al. demonstrated that 80 mg/kg intraperitoneal ketamine twice daily for four days induced CYP1A, CYP2B, CYPE1 and CYP3A proteins in rats by 2-, 13-, 2-, and 2-fold, respectively [20]. CYP2B was, thus, the most responsive to the inductive effect. In line with these results, Lin et al. observed the induction of CYP1A2, CYP3A4, and CYP2B6 in rats after the continuous intragastric administration of 50 mg/kg ketamine for 14 days [21].

Although the doses and intraperitoneal route of administration of ketamine used in these animal models are not comparable to those employed for antidepressant effects in humans, evidence of auto-induction has also been observed in human studies using more representative dosing regimens. Glue et al. investigated the pharmacokinetics of ascending doses of oral, controlled-release, racemic ketamine tablets in healthy subjects [22]. A single dose of 60, 120, or 240 mg or placebo was administered, followed by another five doses every 12 h. The mean area under the curve (AUC) values of both ketamine and norketamine were smaller after multiple dosing when compared to the single-dose mean AUC values in the 120 and 240 mg groups. In patients with major depressive disorder (MDD), an open label, flexible-dose pilot study of the same group including seven patients showed an increased norketamine-to-ketamine ratio after four days of repeated oral ketamine administration, which suggests the occurrence of auto-induction [23].

In addition to the attenuation of efficacy and side effects in long-term treatment for depression, evidence for the development of tolerance to racemic ketamine and esketamine has been described in various other contexts, including anesthesia, pain management, and recreational settings. Several case reports describe tolerance after daily or alternate-day administration of ketamine for anesthesia and with 3–4 times daily administration for chronic pain [24,25,26]. A patient with burn injuries undergoing alternate-day dressing changes with ketamine anesthesia initially received 100 mg IV ketamine, providing 12 min of anesthesia [24]. By the 20th session, recovery of anesthesia occurred after four minutes, and the dose needed to be increased every session. Villanueva-Perez et al. reported administration of 30 mg oral ketamine every eight hours to a patient with complex regional pain syndrome (CRPS), gradually increasing the dose by 5 mg per week to a maximum of 60 mg every six hours [26]. This regimen was maintained for over two years, with significant improvement, particularly during the first four to five months. It is unclear whether the need for higher doses was caused by tolerance development or progression of the CRPS.

In addition to these cases, reports on ketamine abusers describe substantial tolerance, with daily doses reaching up to seven grams per day [27,28,29,30,31]. A patient reported by Pal et al. increased his self-administered dosages of 100–200 mg intramuscular ketamine daily in three to four sessions to approximately 1 g daily over six or seven injections, over a period of two to three months [27]. Goyal et al. report on an anesthesiologist developing misuse and dependence on ketamine, with maximum doses of 2.5 g intramuscular ketamine daily [28]. Two additional cases of ketamine dependence in anesthesia providers were described [29,30]. Both of them stated they had to increase dosage and frequency to achieve similar effects after several months of use. Intranasal ketamine up to 7 g daily was described in a patient who had been using ketamine for six years [29]. This patient reported a rapid escalation in ketamine dosage, describing tolerance development on an hour-by-hour basis, necessitating increasingly higher doses within a single day to achieve the desired effects. After a brief period of abstinence (one day), a substantially lower dose was sufficient to achieve acute intoxication. A study on 65 treatment-seeking patients with ketamine dependence also describe a maximum daily dose (over the past 30 days) of 7 g [31].

It should be noted that, in addition to a pharmacokinetic process (auto-induction), the possibility of the development of tolerance due to pharmacodynamic (neuroadaptive) changes cannot be excluded.

The occurrence of auto-induction could potentially contribute to the attenuation of perceptual and dissociative side effects, as well as reduced therapeutic efficacy in long-term maintenance treatment with ketamine and esketamine. However, treatment regimens with less frequent (e.g., twice weekly) dosing may be less susceptible to auto-induction than daily dosing. Because of better effectiveness, we adapted our study and off-label treatment protocols from daily to twice weekly dosing, mirroring the currently most common clinical practice of (es)ketamine administration at a maximum frequency of twice weekly [5,32,33]. Also, the route of administration may influence the potential process of auto-induction. For instance, intravenous administration bypasses first-pass metabolism. The oral route of delivery includes extensive pre-systemic (gut) and first-pass metabolism, leading to heightened exposure of esketamine to hepatic CYP enzymes, which may accelerate and aggravate the process of auto-induction. Intranasal administration is subject to a mixed scenario, as approximately 50% of the dose is swallowed and metabolized similar to oral esketamine, while the remainder is absorbed directly into systemic circulation through the nasal mucosa.

Although our study was performed in a well-defined sample of TRD patients, it has several limitations, including the small sample size and limited sampling. Due to the relatively small sample size, it was not feasible to perform subgroup analyses based on demographic variables such as age and gender. Given that pharmacokinetics may vary across age groups and between sexes (for instance, as a result of differences in hepatic blood flow), it would be informative to explore the influences of these variables in larger cohorts [34]. Moreover, genetic factors, such as CYP450 polymorphisms, perhaps modulate the extent of auto-induction across patients. For example, genetic polymorphisms of CYP1A2 have been shown to influence the induction activity by omeprazole in healthy subjects [35]. Another limitation is that the model was developed using samples collected on day five, by which time some induction might have already occurred. This could mean that the effect might have been more pronounced if the samples had been collected earlier. In addition, we did not account for the possible effects of hormonal factors. For instance, the menstrual cycle and menopausal status might affect the observed (nor)esketamine concentrations, as E2 and progesterone can induce CYP2B6, CYP3A4, and CYP2A6 [36].

## 4. Materials and Methods

### 4.1. Design

This study was a secondary analysis of an RCT investigating the efficacy of oral esketamine versus placebo in individuals with TRD [32]. The original study was conducted in accordance with the principles outlined in the Declaration of Helsinki. The RCT was approved on 29 August 2016 by the Medical Ethics Review Committee of the University Medical Center Groningen (UMCG) in the Netherlands (file number M16.198879) and registered in the Dutch Trial Register (trial number NTR6161). A detailed study protocol, and the primary outcomes of the RCT, have previously been published [5,32].

**Figure 3:**
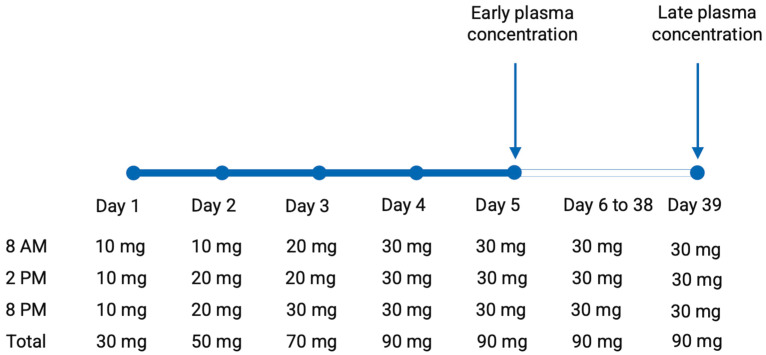
Timeline of the esketamine dosing regimen and the assessments of esketamine and noresketamine plasma concentrations.

In the RCT, participants received oral esketamine thrice daily, as shown in Figure 1. Esketamine doses were administered at 8 AM, 2 PM, and 8 PM. This dosing schedule was chosen to allow for consistent 6-h intervals between doses, while avoiding nocturnal dosing to prevent any possible interference with sleep. Dosages were gradually increased over the first three days and tapered during the last three days, starting on day 40 of treatment (Figure 3). Esketamine and noresketamine plasma concentrations were measured at day 5 of treatment (0, 2, 4, and 6 h post-dose) and at day 39 of treatment (variable time point of assessment). Plasma concentrations were determined using a validated Liquid Chromatography–Tandem Mass Spectrometry (LC tandem MS) assay at the UMCG.

To investigate the potential occurrence of auto-induction in oral esketamine treatment, we developed a population pharmacokinetic model for esketamine and noresketamine. Using this model, we compared the observed plasma concentrations of esketamine and noresketamine measured on day 39 of treatment with the plasma concentrations as predicted by the model at the timepoint of assessment. Details of the population pharmacokinetic model are described in Supplement S1.

### 4.2. Participants

Participants were aged 18 to 80 years and met the Diagnostic and Statistical Manual of Mental Disorders (DSM)-5 criteria for a current episode of unipolar major depressive disorder [37]. All participants had TRD, defined as lifetime non-response to at least three classes of antidepressant medications administered at adequate doses for a minimum of four weeks [5]. Participants also had a Hamilton Depression Rating Scale (HAM-D-17) score greater than 18, indicating moderate to severe depression [38]. All participants were on stable, therapeutic doses of antidepressant medication.

Exclusion criteria were a history of psychotic disorders, bipolar disorder, substance use disorder, and recent use of non-prescribed psychoactive substances or benzodiazepines exceeding 2 mg of lorazepam (or equivalent) per day. Detailed inclusion and exclusion criteria are outlined in the study protocol [5]. Written informed consent was obtained from all participants, who were treated at the UMCG, Pro Persona (Nijmegen), and PsyQ (The Hague). For the present analysis, participants were included only if esketamine and noresketamine plasma concentrations were measured on both day 5 and day 39 of treatment. Of the 113 participants who were randomized, 57 participants were allocated to the esketamine arm. Esketamine plasma concentrations were obtained on both day 5 and day 39 in only a subset of participants, partially since blood sampling was only feasible at two of the three study sites, and partially because of COVID-19-related restrictions during the study period.

### 4.3. Development of a Population Pharmacokinetic Model

Due to limited sampling, we sought existing pharmacokinetic models of esketamine and evaluated these using data from both the original RCT and from an off-label treatment program using oral esketamine [32,33]. In this off-label program, patients with TRD received an individually titrated dosage with a maximum of 3.5 mg/kg of oral esketamine twice weekly over six weeks. A PubMed search was conducted using the following string: (ketamine) AND (pharmacokinetic model)) AND (metabolite)) AND (human)) AND (population) (1 June 2024). This yielded 15 studies, 5 of which presented pharmacokinetic models [39,40,41,42,43]. Among these, the model for intravenous esketamine, developed by Kamp et al. (2020) [39], was adapted to incorporate the absorption rate and first-pass metabolism for oral administration. This adapted model, developed using the population pharmacokinetic modeling software Edsim++ (version 2.5.0.154) [44,45,46], provided the best relative fit to our data, in comparison to the other pharmacokinetic models described in the literature (see Supplement S1). Consequently, this modified model was used to predict esketamine and noresketamine concentrations on day 39 of treatment. A detailed description of the development of our population pharmacokinetic model is provided in Supplement S1.

### 4.4. Statistical Analysis

Esketamine and noresketamine plasma concentrations on day 39 of oral esketamine treatment, as measured in the original RCT and as predicted by our model, were assessed and compared. First, Shapiro–Wilk tests were conducted to examine if the differences between measured and predicted esketamine and noresketamine plasma concentrations were normally distributed. Subsequently, measured and predicted esketamine and noresketamine were compared using paired *t*-tests (one-sided). Post hoc, we investigated whether patients with the greatest difference between measured and predicted esketamine concentrations also had the greatest difference between measured and predicted noresketamine concentrations, using a Pearson correlation. Statistical significance was defined as *p* ≤ 0.05. All statistical analyses were conducted using SPSS version 30 [47].

## 5. Conclusions

In this study, we developed a population pharmacokinetic model for oral esketamine and found evidence suggesting the occurrence of auto-induction during prolonged thrice-daily dosing. The observed lower-than-predicted plasma concentrations of both esketamine and noresketamine support the hypothesis that enzyme induction—likely involving CYP3A4 and CYP2B6—may alter esketamine pharmacokinetics over time. Our findings are consistent with the results of previous preclinical and clinical studies and may help explain the development of tolerance to (es)ketamine during frequent use.

Given the present study’s limitations—including the small sample size and limited sampling time points—further research in larger patient populations and with multiple data collection time points is warranted. In addition, future studies could investigate any influence of genetic variations in CYP enzyme activity or the possible effects of hormonal factors.

Moreover, it would be relevant to assess whether lower esketamine and noresketamine plasma concentrations indeed lead to diminished antidepressant effects. If so, personalized therapeutic strategies may be needed, such as dose adjustments, less frequent administration, treatment pauses (“drug holidays”), or incorporating CYP enzyme inhibitors to maintain therapeutic effects [48].

## Data Availability

Access to individual-level data is restricted only to individuals named in the study permit.

## Supplementary Materials

The following supporting information can be downloaded at: https://www.mdpi.com/article/10.3390/ph18050627/s1, Supplement S1. Development of a population pharmacokinetic model.
