# Supplementary material for: Auto-Induction in Oral Esketamine Treatment for Treatment-Resistant Depression: An Exploratory Study
Source: Pharmaceuticals (Basel). 2025 Apr 25;18(5):627. doi: 10.3390/ph18050627 (PMC12114491; doi:10.3390/ph18050627)
Supplement: Supplementary file 1 [file pharmaceuticals-18-00627-s001.zip › pharmaceuticals-3544602-supplementary.pdf]

Supplement S1: Development of a population pharmacokinetic model

Due to limited sampling, we sought existing pharmacokinetic models of esketamine and evaluated these using data from the original RCT and from an off-label trial using oral esketamine administration (32). In this off-label trial, patients received a maximum of 3.5 mg/kg of oral esketamine twice weekly over six weeks.

A PubMed search was conducted using the following string: (ketamine) AND (pharmacokinetic model)) AND (metabolite)) AND (human)) AND (population) (1 June 2024). This yielded 15 studies, five of which presented pharmacokinetic models, as summarized in Table A.

Table A. Population pharmacokinetic models for esketamine.

First author, year	Compartments	Route of administration
Kamp, 2020 (39)	Esketamine: 2<br>Noresketamine: 2	Intravenous
Fanta, 2015 (40)	Esketamine: 3<br>Noresketamine: 3	Intravenous, oral
Zhao, 2012 (41)	Esketamine: 3<br>Noresketamine: 2	Intravenous
Weiss, 2022 (42)	Esketamine: 3<br>Noresketamine: 2	Intravenous
Perez-Ruixo, 2021 (43)	Esketamine: 3<br>Noresketamine: 2	Nasal, oral, intravenous

Among the available models, the Fanta model [40] was selected for implementation in Edsim++ (version 2.5.0.154) [44-46]. This selection was based on the inclusion of oral administration in the Fanta model and its linear form. Although the Perez-Ruixo model [43] also included oral administration, the Fanta model was preferred due to its ease of adaptation. However, during implementation, it became

apparent that the model was unable to accurately predict the concentration curves. Consequently, the decision was made to transition from the Fanta model to the Kamp model [39]. The Kamp model effectively predicted the concentrations of both esketamine and noresketamine using two separate two-compartment models, connected by two metabolism compartments.

However, the Kamp model did not support oral administration. To address this limitation, oral administration and first-pass metabolism were incorporated into the existing model. Furthermore, the peripheral hydroxynorketamine (HNK) compartment was removed to ensure a more concise model.

The Kamp model assumes complete metabolism of esketamine into noresketamine, with similar absorption kinetics for both compounds in the gut. Implementation in Edsim++ commenced with an XSplitter, which divided the dose into bioavailable esketamine (F) and a fraction directly converted to noresketamine (1-F). Esketamine was modeled to pass through an initial compartment before entering the central esketamine compartment, represented in Edsim++ by a TCompartment object connected to a TTransport object. This was followed by two compartments for esketamine distribution and elimination, in accordance with Kamp's model. Metabolism into noresketamine was modeled using a TMetabolism object linked to a TCompartment object representing the liver. From the liver compartment, esketamine was transported into the central noresketamine compartment, modeled in Edsim++ using a TTransit object connecting both the liver and central noresketamine compartments.

The absorption pathway of noresketamine was modeled as direct absorption into the liver compartment described above. This was implemented in Edsim++ by linking the XSplitter object to the TCompartment representing the liver after esketamine metabolism. Noresketamine was subsequently distributed into two peripheral compartments and eliminated from the central compartment via a TElimination object. Following its elimination, noresketamine undergoes metabolism into both HNK and dehydronorketamine (DHNK), a process modeled in Edsim++ using a TMetabolismBi object. One end of this object was connected to the central HNK compartment, while the other was linked to a

second liver compartment. HNK was then absorbed into the central compartment and eliminated through a TElimination object. DHNK, entered the second liver compartment, modeled as a TCompartment object. From this liver compartment, DHNK was transported to the central from which it is then eliminated. This process was modeled in Edsim++ by linking a TTransport object to the liver compartment, followed by a connection between the TTransport object and the central DHNK compartment, which was then linked to a TElimination object. The central compartments for norketamine, HNK, and DHNK were modeled as XCompartment objects, as they shared the same parameters as the central esketamine compartment.

To support model fitting, an Excel dataset was compiled, incorporating data from an off-label study and the RCT. The dataset included patient characteristics, dosing regimens, and measured plasma concentrations. Blood samples collected after six weeks were excluded to optimize the computational efficiency of pharmacokinetic parameter fitting. Bayesian fitting was employed due to the limited number of data points per patient, using Kinpop, an Edsim++ extension. Kinpop iteratively refined population parameters by fitting them to individual patient data until the optimal fit was achieved. Settings were adjusted to a population-fixed Bayes approach, ensuring consistent fitting across all patients.

The adapted pharmacokinetic model successfully extended the existing intravenous esketamine model by Kamp et al. (2020) to accommodate oral administration (39).
